# Supplementary material for: Effect of topical emollient oil application on weight of preterm newborns: A systematic review and meta-analysis
Source: PLoS One. 2024 May 14;19(5):e0302969. doi: 10.1371/journal.pone.0302969 (PMC11093394; doi:10.1371/journal.pone.0302969)
Supplement: S2 Appendix — (PDF) [file pone.0302969.s002.pdf]

Appendix 2:- Search Strategies Queries Results

S. No	Data base	Searching Combination	# article (Total record)	Searching date	Remark
1	Cochrane	Emollient* OR "sunflower oil" OR "Coconut oil" OR "Soybean Oil" OR "olive oil" OR ISIO4 OR Aquaphor* OR Oil in Title Abstract Keyword AND "Preterm Infant" OR "premature infant" OR preterm OR "Infant, Premature" OR "Premature Birth" in Title Abstract Keyword	411	July 13, 2023	
2	Clinical trials	Each searching terms, With additional filter (interventional, child up to17 years)	73	July 13, 2023	
3	PubMed	(("emollient*"[Title/Abstract] OR "emollient*"[MeSH Terms] OR "sunflower oil"[Title/Abstract] OR "Coconut oil"[Title/Abstract] OR "Soybean Oil"[Title/Abstract] OR "olive oil"[Title/Abstract] OR "ISIO4"[Title/Abstract] OR "aquaphor*"[Title/Abstract] OR "Oil"[Title/Abstract]) AND ("Preterm Infant"[Title/Abstract] OR "premature infant"[Title/Abstract] OR "preterm"[Title/Abstract] OR "infant, premature"[MeSH Terms] OR "Premature Birth"[MeSH Terms]))	449	July 12, 2023	***Additional filters*** (publication date = 2000/1/1:2023/7/13)
4	Scopus	TITLE-ABS-KEY (emollient* OR "sunflower oil" OR "Coconut oil" OR "Soybean Oil" OR "olive oil" OR isio4 OR aquaphor* OR oil) AND TITLE-ABS-KEY ("Preterm Infant" OR "premature infant" OR preterm OR "Premature Birth")	698	July 12, 2023	***Additional filters*** (publication date = 2000 - 2023)
5	ProQuest Central	noft(emollient* OR "sunflower oil" OR "Coconut oil" OR "Soybean Oil" OR "olive oil" OR isio4 OR aquaphor* OR oil) AND noft("Preterm Infant" OR "premature infant" OR preterm OR "Premature Birth")	479	July 12, 2023	***Additional filters*** (publication date = 2000 - 2023)
6	Epistemonikos	(title:(emollient*) OR abstract:(emollient*)) OR (title:("emollient oil") OR abstract:("emollient oil")) OR (title:("sunflower oil") OR abstract:("sunflower oil")) OR (title:("Coconut oil") OR abstract:("Coconut oil")) OR (title:("Soybean Oil") OR abstract:("Soybean Oil")) OR (title:("olive oil") OR abstract:("olive oil")) OR (title:(ISIO4) OR abstract:(ISIO4)) OR (title:(Aquaphor*) OR abstract:(Aquaphor*)) OR (title:(Oil) OR abstract:(Oil)) AND (title:("Preterm Infant") OR abstract:("Preterm Infant")) OR (title:("premature infant") OR abstract:("premature infant")) OR (title:("Preterm neonate") OR abstract:("Preterm neonate")) OR (title:("Preterm newborn") OR abstract:("Preterm newborn")) OR (title:(preterm) OR abstract:(preterm))	357	July 13, 2023	***Additional filters*** (publication year = 2000 – 2023, publication type = primary study, study design = RCT)
**Subtotal from databases**			**2467**		

7	Hinari	emollient oil application on preterm	31	July 13, 2023	
8	Science direct	emollient oil application on preterm	29	July 13, 2023	(research articles)
9	Google scholar	effect of emollient oil application on preterm neonate randomized control trial	114	July 8, 2023	
10	Global Index Medicus	(tw:(emollient* OR "sunflower oil" OR "Coconut oil" OR "Soybean Oil" OR "olive oil" OR isio4 OR aquaphor* OR oil)) AND (tw:("Preterm Infant" OR "premature infant" OR preterm OR "Premature Birth"))	11	July 13, 2023	out of 37 articles
11	Wiley Online Library	effect of emollient oil application in preterm neonate randomized control trial	28	July 7, 2023	
12	Mednar	effect of emollient oil application on preterm neonate randomized control trial	24	July 12, 2023	
13	World cat	ti:emollient* OR ti:"sunflower oil" OR ti:"Coconut oil" OR ti:oil AND ti:preterm infant	18	July 12, 2023	
14	Across references		12		
**Subtotal from other sources**			**267**		
**Total number of retrieved articles**			**2,734**		
