# Supplementary material for: Effect of topical emollient oil application on weight of preterm newborns: A systematic review and meta-analysis
Source: PLoS One. 2024 May 14;19(5):e0302969. doi: 10.1371/journal.pone.0302969 (PMC11093394; doi:10.1371/journal.pone.0302969)
Supplement: S3 Appendix — (PDF) [file pone.0302969.s003.pdf]

Appendix 3: - List of excluded studies from final analysis of pooled difference in mean weight on effect of topical emollient oil application on weight gain of preterm neonate, 2023

S.no	Title	URL or Link
1.	Sunflower seed oil and infection	https://www.proquest.com/trade-journals/sunflowerseed-oil-infection/docview/223610263/se-2?accountid=17194%20%20%20%20%20https://libkey.io/libraries/2954/openurl?genre=article&au=Anonymous&aulast=Anonymous&issn=15289303&isbn=&title=Sunflowerseed+oil+and+infection&jtitle=INFORM&pubname=INFORM&btitle=&atitle=Sunflowerseed+oil+and+infection&volume=16&issue=5&spage=297&date=2005&doi=&sid=ProQuest%20%20%20%20%20https://media.proquest.com/media/hms/OBJ/TEujS?_a=ChgyMDIzMDcxMTIxMTA1MDQ2MjoyMDAxNjkSBTkxMjExGgpPTkVfU0VBUkNIIgoxNjQuMTUuMS4xKgUzNzEzMTIJMjIzNjEwMjYzOg1Eb2N1bWVudEltYWdlQgEwUgZPbmxpbmVaAkZUYgNQRlRqCjIwMDUvMDUvMDFyCjIwMDUvMDUvMzF6AIIBKVAtMTAwMDI4My0xNzE5NC1DVVNUT01FUi0xMDAwMDI1NS00NzMyMjYykgEGT25saW5lygFvTW96aWxsYS81LjAgKFdpbmRvd3MgTlQgMTAuMDsgV2luNjQ7IHg2NCkgQXBwbGVXZWJLaXQvNTM3LjM2IChLSFRNTCwgbGlrZSBHZWNrbykgQ2hyb21lLzExNC4wLjAuMCBTYWZhcmkvNTM3LjM20gEOVHJhZGUgSm91cm5hbHOaAgdQcmVQYWlkqgIrT1M6RU1TLU1lZGlhTGlua3NTZXJ2aWNlLWdldE1lZGlhVXJsRm9ySXRlbcoCBE5ld3PSAgFZ8gIA%2BgIBToIDA1dlYooDHENJRDoyMDIzMDcxMTIxMTA1MDQ2Mjo3ODc0NTQ%3D&_s=qrLtJfHfW1G6hg1OVOIjrS%2BgbfI%3D
2.	Olive Oil Massage Effect for Reduction of Preterm Sepsis (OMEPS)	https://www.proquest.com/wire-feeds/clinical-trial-olive-oil-massage-effect-reduction/docview/2479218619/se-2?accountid=17194%20%20%20%20%20https://libkey.io/libraries/2954/openurl?genre=article&au=&aulast=&issn=&isbn=&title=Clinical+Trial%3A+Olive+Oil+Massage+Effect+for+Reduction+of+Preterm+Sepsis+%28OMEPS%29&jtitle=US+Fed+News+Service%2C+Including+US+State+News&pubname=US+Fed+News+Service%2C+Including+US+State+News&btitle=&atitle=Clinical+Trial%3A+Olive+Oil+Massage+Effect+for+Reduction+of+Preterm+Sepsis+%28OMEPS%29&volume=&issue=&spage=&date=2021&doi=&sid=ProQuest
3.	The Effect of Skin Care Applied With Two Different Oils on Skin Integrity and Growth Parameters in Premature Babies	https://www.proquest.com/wire-feeds/clinical-trial-effect-skin-care-applied-with-two/docview/2554186450/se-2?accountid=17194%20%20%20%20%20https://libkey.io/libraries/2954/openurl?genre=article&au=&aulast=&issn=&isbn=&title=Clinical+Trial%3A+The+Effect+of+Skin+Care+Applied+With+Two+Different+Oils+on+Skin+Integrity+and+Growth+Parameters+in+Premature+Babies&jtitle=US+Fed+News+Service%2C+Including+US+State+News&pubname=US+Fed+News+Service%2C+Including+US+State+News&btitle=&atitle=Clinical+Trial%3A+The+Effect+of+Skin+Care+Applied+With+Two+Different+Oils+on+Skin+Integrity+and+Growth+Parameters+in+Premature+Babies&volume=&issue=&spage=&date=2021&doi=&sid=ProQuest

4.	Effect of topical sunflower oil in preventing nosocomial infections in premature infants	**https://www.researchgate.net/publication/330657944_Effect_of_topical_sunflower_oil_in_preventing_nosocomial_infections_in_premature_infants**
5.	Emollient Therapy In Preterm & Low Birth Weight Neonates: A Randomized Clinical Trial	**https://www.proquest.com/wire-feeds/clinical-trial-emollient-therapy-preterm-amp-low/docview/2458224526/se-2?accountid=17194%20%20%20%20%20https://libkey.io/libraries/2954/openurl?genre=article&au=&aulast=&issn=&isbn=&title=Clinical+Trial%3A+Emollient+Therapy+In+Preterm+%26amp%3B+Low+Birth+Weight+Neonates%3A+A+Randomized+Clinical+Trial&jtitle=US+Fed+News+Service%2C+Including+US+State+News&pubname=US+Fed+News+Service%2C+Including+US+State+News&btitle=&atitle=Clinical+Trial%3A+Emollient+Therapy+In+Preterm+%26amp%3B+Low+Birth+Weight+Neonates%3A+A+Randomized+Clinical+Trial&volume=&issue=&spage=&date=2020&doi=&sid=ProQuest**
6.	Efficacy and Safety of Virgin Coconut Oil as an Adjunct in the Care of Premature Infants in a Tertiary Medical Center: A Randomized, Controlled, Clinical trial.	Efficacy and Safety of Virgin Coconut Oil as an Adjunct in the Care of Premature Infants in a Tertiary Medical Center: A Randomized, Controlled, Clinical trial. | HERDIN
