# Supplementary material for: Effect of topical emollient oil application on weight of preterm newborns: A systematic review and meta-analysis
Source: PLoS One. 2024 May 14;19(5):e0302969. doi: 10.1371/journal.pone.0302969 (PMC11093394; doi:10.1371/journal.pone.0302969)
Supplement: S4 Appendix — (PDF) [file pone.0302969.s004.pdf]

Appendix 4: - Quality Assessment Based on Cochrane Risk of Bias Tool for RCTs 'ROB 2'

First Author name, Year	Country	Bias arising from the randomization process	Blinding of Participants and Personnel	Incomplete Outcome	Blinding of Outcome Assessment	Selective reporting	Overall risk of bias Judgment
Khatun N, 2021 (16)	India	Low risk	Low risk	Low risk	Low risk	Low risk	Low risk
Liao Y, 2021 (13)	Taiwan	Low risk	Low risk	Low risk	Low risk	Low risk	Low risk
Oriot D, 2008 (37)	France	Low risk	Low risk	Low risk	Low risk	Low risk	Low risk
Gonzalez AP, 2009 (35)	Mexico	Low risk	Low risk	Low risk	Low risk	Low risk	Low risk
Montaseri S, 2020 (30)	Iran	Low risk	Low risk	Low risk	Low risk	Low risk	Low risk
Fallah, Razieh, 2013 (31)	Iran	Low risk	Low risk	Low risk	Low risk	Low risk	Low risk
Salam RA, 2015 (29)	Pakistan	Low risk	Low risk	Low risk	Low risk	Low risk	Low risk
Soriano R, 2000 (36)	Brazil	Low risk	Low risk	Low risk	Low risk	Low risk	Low risk
Armand M, 2022 (38)	France	Low risk	Low risk	Low risk	Some concern	Low risk	Some concerns
Jabraeile M, 2016 (33)	Iran	Some concerns	Low risk	Low risk	Low risk	Low risk	Some concerns
Saeidi, 2009 (26)	Iran	Some concern	Low risk	Low risk	Low risk	Low risk	Some concerns
Kumar and Upadhyay, 2013(23)	India	Low risk	Low risk	Low risk	Some concerns	Low risk	Some concerns
Saeadi, Reza, 2015 (25)	Iran	Some concern	Some concern	Low risk	Some concern	Low risk	High risk
Jamshaid AA, 2021 (24)	Pakistan	Some concern	Some concern	Low risk	Some concern	Low risk	High risk
Arora J, 2005 (32)	India	Low risk	Some concern	Low risk	Some concern	Low risk	High risk
K. Sankaranarayanan, 2005 (28)	India	Low risk	Some concern	Low risk	Some concern	Low risk	High risk
Strunk T, 2017 (34)	Australia	Low risk	Some concern	Low risk	Low risk	Some concerns	High risk
Al-Abdullah, 2012 (27)	Saudi Arabia	High risk	Some concern	Low risk	Some concern	Low risk	High risk
