# Supplementary material for: Effect of topical emollient oil application on weight of preterm newborns: A systematic review and meta-analysis
Source: PLoS One. 2024 May 14;19(5):e0302969. doi: 10.1371/journal.pone.0302969 (PMC11093394; doi:10.1371/journal.pone.0302969)
Supplement: Attachment — Submitted filename: Point by Point response for reviwer.pdf [file pone.0302969.s005.pdf]

Dear Editors and Reviewers,

We would like to express our sincere appreciation for your dedicated effort in critically reviewing our work. Your valuable feedback has played a pivotal role in enhancing the quality of our manuscript and ensuring the robustness and significance of the study's findings.

Responses for Reviewer One

1. There was a published systematic review by Rehana AS et al., 2013 (DOI: 10.1186/1471-2458-13-S3-S31) that mentioned the efficacy of emollient therapy on newborns' weight gain. The authors of this study should justify how this review would generate additional evidence and value over the previous article by Rehana AS et al.

Response: Thank you for your valuable suggestions. We acknowledge the previous study conducted by Rehana AS et al. and appreciate their contributions to the field. Their study focused specifically on research conducted in the developing world and included data up until December 2012. Furthermore, their analysis was limited to four studies with a sample size of 300, primarily examining the weight difference among groups.

In contrast, our study aims to build upon their research by providing an updated and comprehensive analysis of the impact of emollient oil application on weight gain in preterm newborns. We have expanded the scope and timeframe of our analysis to include studies conducted worldwide until 2023. This broader approach has allowed us to incorporate a larger sample size of 1,406 participants, enabling us to capture a more diverse range of studies and enhance the statistical power of our findings.

2. The authors mentioned that the rationale of this study is to synthesise current evidence on interventions to improve survival outcomes in preterm infants, and emollient therapy would be one of the important management strategies. However, based on existing evidence of the beneficial effects of emollient therapy, the WHO has already recommended the application of 'natural' oils (e.g. sunflower and coconut oil) to the body of preterm or LBW infants to increase weight gain (DOI: 10.1016/j.eclinm.2023.102155). Priority for further research has been set to "Which emollients (which oils, which composition) are most effective and safe? And their optimal regime (dose, frequency, duration) and mode of application" (DOI: 0.1016/j.eclinm.2023.102126). Thus, the authors should describe strong arguments on how the findings of this review study add further value to the scientific community.

Response: Thank you for your concerns and suggestions. Despite the recommendation by the World Health Organization (WHO) regarding the application of emollient oil in preterm and low birth weight babies, there still exists a controversy among the results reported in the literature. Our study aims to address these controversies and provide valuable insights into the use of emollient oils in this population.

Moreover, our study goes beyond examining the overall impact of emollient oil application. We also investigate important factors such as the specific type of emollient oil used, the frequency and dosage of application, as well as potential adverse events associated with its use. By addressing these aspects, our research contributes to enhancing the practical application of this intervention in day-to-day clinical practice.

Furthermore, another crucial aspect of our study is the identification of research gaps and areas of uncertainty. By highlighting these gaps, we aim to guide and inspire further research in this field. This comprehensive approach not only provides valuable insights for clinical decision-making but also serves as a foundation for future investigations.

We have made a concerted effort to include these significant points in the main document, ensuring that the implications of our study are clearly communicated.

3. The authors concluded with that "Therefore, Policymakers and health planners should give great emphasis on implementation of the use of emollient oils in the routine care of preterm infants to assist with weight gain." As per my previous comment, the policy makers (e.g. WHO) has already considered and recommended emollient therapy in to a standard management therapy. The author should mention what further benefit can be achieved from this study.

Response: I completely agree with your point. Despite the WHO recommendation for the application of emollient oil in preterm and low birth weight babies, it is often overlooked or not incorporated into treatment or management guidelines in different countries, including Ethiopia as an example.

The findings of our study, which provide insights into the best type of emollient oil, appropriate dosage, frequency, duration, and potential adverse events associated with its application, highlight the importance of local policy makers taking action. In the Ethiopian context, for instance, it is vital for the Ministry of Health and other relevant stakeholders to support the implementation of emollient oil application as a routine care strategy for preterm infants.

By incorporating this evidence-based intervention into local guidelines and policies, healthcare providers can enhance the care provided to preterm infants, potentially leading to improved outcomes and reduced complications. It is crucial to advocate for the inclusion of emollient oil application as a standard practice, ensuring that preterm infants receive the benefits of this intervention as part of their routine care.

4. The study included 17 articles published between 2000 to July 2023. But I wonder if they missed more important RCTs on this topic that were published in this time frame. For example, Kumar V et al, 2022 (doi: 10.1093/ajcn/nqab430.); Sankaranarayanan K, et al., 2005, PMID: 16208048; Kumar J et al, 2012 (DOI: 10.1007/s12098-012-0869-7). Authors should revise their search and include all missed articles.

Response: Apologies for the confusion caused by the citation issue. We would like to clarify that the study conducted by K. Sankaranarayanan was indeed included in our final analysis. The problem arose with the citation, as the corresponding author's name, Mankir, was mistakenly listed as the first author. However, we have rectified this error in the main document by providing the accurate and corrected citation.

Regarding the study by Kumar V et al., 2022, it was appropriately excluded during the full-text review because it involved term babies and compared the efficacy of sunflower oil versus mustard oil, which did not align with our study's focus on routine care or no oil application in preterm infants. Therefore, it did not meet our strict eligibility criteria.

However, we want to acknowledge that we made an oversight in initially excluding the study conducted by Kumar J et al., 2012 from our final analysis. We take full responsibility for this error, and we have now incorporated it as the eighteenth study in our analysis. By reanalyzing the entire analysis section, we ensure that our findings are robust, comprehensive, and reflect the complete body of relevant literature.

We genuinely appreciate your critical review of our work, as it has helped us identify and address these issues.

5. The authors did the pooled analysis on the mean weight gain after emollient therapy, and it showed significant results. However, their studies were highly heterogeneous. They differ in terms of emollient type, duration and dose. Thus, it may reduce the strength of the study results. Although the authors did subgroup analysis on the pooled analysis, the sample size is too low

to strengthen the results. For example, the coconut oil group had two studies, 5ml/kg/day oil group had two studies.

Response: Thank you for highlighting the issue of heterogeneity in the studies included in our analysis. We fully acknowledge this concern, and to address it, we conducted sensitivity analyses, subgroup analyses, and meta-regression. These additional analyses aimed to explore potential sources of heterogeneity and provide a more nuanced understanding of the findings. Furthermore, in light of the heterogeneity observed, we have included recommendations for future research in our study. We emphasize the need for well-designed randomized controlled trials that adhere to standardized protocols.

6. The authors did a quality evaluation of the studies by using the Cochrane Risk of Bias Tool for Randomized Controlled Trials (Higgins et al., 2011), which is an old tool. The 'RoB 2' has been revised in August 2019. So, the author should use the updated tool for a better acceptability of their methodology.

Response: We greatly appreciate your valuable feedback. Taking into consideration your insightful suggestions, we have already taken the necessary steps to update our risk of bias assessment tool to the 'RoB 2' version of the Cochrane Risk of Bias Tool for RCTs.

7. As the authors mentioned, the weight gain is multifactorial. Yes, it depends on the combination of the type of emollient oil, its dose and duration. Pooled analysis on homogenous methodological studies can further refute the findings of this study. Thus, research priority has been set to assess the efficacy of the best emollient oil across all available oils for a specific dose and duration. The authors should describe this point, describe the weakness of the heterogeneous study analysis, and come up with some recommendations for future RCTs.

Response: We sincerely value your valuable feedback and suggestions. Based on your insightful input, we have made significant revisions to our manuscript, specifically heterogeneity of studies and the need for further research.

Responses for Reviewer two

1. The authors of 'Effect of Topical Emollient Oil Application on Weight Gain of Preterm Newborns: A Systematic Review and Meta-Analysis' have worked on a relevant topic and the review is well performed but my major concern is that this would need thorough language correction and editing.

Response: We would like to express our gratitude for recognizing the relevance and quality of our work. We highly appreciate your positive feedback.
We acknowledge your valid concern regarding the need for thorough language correction and editing in our manuscript. We want to assure you that we have made diligent efforts to address this issue. We have taken your feedback into careful consideration and have allocated sufficient time and resources to perform a comprehensive language correction and editing process.

2. Other comments
   - ✓ The background does not cite all the previous systematic reviews that have been done for this intervention looking at weight and also other outcomes.
   - ✓ Rationale for this systematic review needs to be strengthened
   - ✓ Methods should start with objectives
   - ✓ The authors should mention that how many authors were contacted for full texts and missing data and how many responded
   - ✓ Was ROB 1 used or ROB 2 used for quality assessment.
   - ✓ Methods should define the planned sub-groups
   - ✓ What was the time or age at which weight was measured?
   - ✓ For conclusion, the authors need to make the clinical relevance of about 50g gain in weight and also mention that over what time this change occurred.

Response: We greatly appreciate your valuable feedback and suggestions. We have carefully reviewed and incorporated all of your insightful comments into our manuscript.
Your input has played a crucial role in improving the overall quality and clarity of our work. We have thoroughly revised the manuscript, addressing each of the concerns and suggestions you raised.
