# Supplementary material for: Effect of topical emollient oil application on weight of preterm newborns: A systematic review and meta-analysis
Source: PLoS One. 2024 May 14;19(5):e0302969. doi: 10.1371/journal.pone.0302969 (PMC11093394; doi:10.1371/journal.pone.0302969)
Supplement: Attachment — Submitted filename: Point by Point response - 2.pdf [file pone.0302969.s006.pdf]

Point By Point response

We would like to express our deepest appreciation to the **editors** for their diligent management of the peer review process. Their careful oversight and guidance have been instrumental in ensuring the integrity and quality of our work. We would also like to extend our heartfelt gratitude to the esteemed **reviewers** who invested their valuable time and expertise in reviewing our papers and providing insightful suggestions. We are grateful for their contributions, and we have thoroughly addressed all their concerns and incorporated their feedback to enhance the overall quality of our research.

Reviewer major concerns:

1. I would like the authors to address one of the comments from the reviewers on the clinical significance of the difference in mean weight of 52 grams from a policy perspective. The reviewer commented that "I would like the authors to still make the clinical relevance of about 50g gain in weight and also mention that over what time this change occurred. This is important before advocating for its recommendation." Authors can discuss this finding in the 'Discussion' section and frame the conclusion accordingly.

   Response: Thank you for your feedback. In the revised Discussion section of our manuscript, we have devoted explicit attention to addressing the clinical significance of weight gain and the associated policy implications. Additionally, within the "Characteristics of Included Studies" section, we have incorporated regarding the duration of time during which the observed weight gain occurred.

2. In the abstract and the methodology section, the authors have referred to the outcome of interest as 'weight gain' rather than 'weight'. I suggest that the authors refer to the outcome of interest as 'weight' since the outcome of interest is 'weight' and not 'weight gain'. 'weight gain' was a finding of the review.

   Response: We appreciate your suggestion, and we have made the necessary revisions to ensure consistent terminology throughout the entire manuscript.

3. The PRISMA checklist requires the authors to report assessments of certainty (or confidence) in the body of evidence for each outcome assessed. However, the authors have not reported

GRADE certainity of the outcomes. I would suggest that the authors assess and report the certainity of evidence for the outcomes of interest.

Response: We have taken your suggestion into consideration and performed a GRADE assessment to evaluate the certainty of evidence for the outcomes of interest. The results of this assessment have been incorporated into the conclusion and discussion section of the revised manuscript.

minor concerns:

Response: We have made thorough efforts to address all the minor concerns raised by the esteemed reviewers. Their feedback was carefully reviewed, and we made the necessary adjustments to the manuscript to ensure that each concern was effectively addressed.

4. Characteristics of included studies: I would suggest that the authors specify the range of the mean gestational age as well as the baseline mean weight of the preterm babies from the included studies. These could potentially add context to the heterogeneity in the results.

Response: Thank you for your concerns. In order to provide clarification on these points, we acknowledge that some of the included studies reported gestational age ranges (e.g., 32-36 weeks) or used terminology such as "<37 weeks," which can limit our ability to calculate precise mean values. Additionally, we understand that certain studies may have only reported the mean weight difference without providing specific baseline weight data for both intervention and comparison groups, making it difficult to include them in our subgroup analysis.

Given these limitations in the available data, we have provided transparency regarding these issues in the "Characteristics of Included Studies" section of the manuscript. We highlight the challenges in computing precise mean gestational age and baseline weight due to the variability in reporting across the included studies.
